# Opportunities and Pitfalls of Fluorescent Labeling Methodologies for Extracellular Vesicle Profiling on High-Resolution Single-Particle Platforms

**DOI:** 10.3390/ijms221910510

**Published:** 2021-09-29

**Authors:** Diogo Fortunato, Danilo Mladenović, Mattia Criscuoli, Francesca Loria, Kadi-Liis Veiman, Davide Zocco, Kairi Koort, Natasa Zarovni

**Affiliations:** 1 Exosomics SpA, 53100 Siena, Italy; dfortunato@exosomics.eu (D.F.); mcriscuoli@exosomics.eu (M.C.); dzocco@exosomics.eu (D.Z.); 2 HansaBioMed Life Sciences Ltd., 12618 Tallinn, Estonia; danilo@hansabiomed.eu (D.M.); francesca@hansabiomed.eu (F.L.); kadi-liis@hansabiomed.eu (K.-L.V.); 3 School of Natural Sciences and Health, Tallinn University, 10120 Tallinn, Estonia; kairi.koort@tlu.ee; 4 Cell and Gene Therapy Research and Development, Lonza Inc., Rockville, MD 20850, USA

**Keywords:** extracellular vesicles, exosomes, flow cytometry, nanoparticle tracking analysis, fluorescent dyes, purification, isolation, subpopulations, tetraspanin, antibody

## Abstract

The relevance of extracellular vesicles (EVs) has grown exponentially, together with innovative basic research branches that feed medical and bioengineering applications. Such attraction has been fostered by the biological roles of EVs, as they carry biomolecules from any cell type to trigger systemic paracrine signaling or to dispose metabolism products. To fulfill their roles, EVs are transported through circulating biofluids, which can be exploited for the administration of therapeutic nanostructures or collected to intercept relevant EV-contained biomarkers. Despite their potential, EVs are ubiquitous and considerably heterogeneous. Therefore, it is fundamental to profile and identify subpopulations of interest. In this study, we optimized EV-labeling protocols on two different high-resolution single-particle platforms, the NanoFCM NanoAnalyzer (nFCM) and Particle Metrix ZetaView Fluorescence Nanoparticle Tracking Analyzer (F-NTA). In addition to the information obtained by particles’ scattered light, purified and non-purified EVs from different cell sources were fluorescently stained with combinations of specific dyes and antibodies to facilitate their identification and characterization. Despite the validity and compatibility of EV-labeling strategies, they should be optimized for each platform. Since EVs can be easily confounded with similar-sized nanoparticles, it is imperative to control instrument settings and the specificity of staining protocols in order to conduct a rigorous and informative analysis.

## 1. Introduction

For the past two decades, EV research has risen exponentially along with the outstanding discoveries that have revealed numerous biological functions mediated or directly executed by EVs. These membrane-enclosed nanoparticles virtually encapsulate any biomolecule type found in respective donor cells, namely: DNA, RNA, proteins, lipids, or metabolites [1,2,3]. Nearly all of the cells in the human body actively secrete EVs, which can circulate through all sorts of biological fluids such as plasma, urine, or saliva [1]. Recently, the scientific community realized that EVs were significantly promising non-invasive indicators of an individual’s global health status. This sparked the race for the discovery of EV-specific biomarkers, with the goal of improving or enabling the detection of a number of diseases and translating novel non-invasive practices into routine clinical use. Since small EVs (sEVs), typically in a nanoscale range (30–200 nm), are highly heterogeneous and indistinguishable from other biological nanoparticles, the absolute need to perform true single-vesicle discrimination, analysis, and characterization quickly emerged. For this purpose, high-resolution methodologies, such as nanoparticle tracking analysis (NTA) [4,5,6,7,8], tunable resistive pulse sensing (TRPS) [9,10], Raman spectroscopy [11,12,13], atomic force microscopy [14,15], super-resolution microscopy [16,17,18,19], or nanoflow cytometry [20,21,22,23,24], have been explored [25,26]. Techniques relying on label-free EV analysis can estimate particle size, concentration, and other physical parameters such as zeta potential. Methods focused exclusively on fluorescence measurements help to determine the nature or composition of labelled particles within the samples. Hence, it becomes clear that approaches incorporating both label-free and fluorescence measurements may provide the necessary robustness to discriminate true sEVs in complex samples and to quantitatively characterize relevant subpopulations, often present in extremely low abundance.

In conventional flow cytometers, light scatter measurements alone enable a high-throughput multiparametric analysis of microscopic particles, which can be combined with fluorescent labeling to pinpoint and characterize specific components or biological processes. However, due to the physical properties of particles and light, conventional flow cytometers fail to detect events under 200 nm [27]. Summarily, the intensity of scattered light decreases by orders of magnitude (sixth power), for particles with size smaller than the wavelength of the incident light [28,29]. In order to accurately detect sEVs, dedicated state-of-the-art systems have been developed to increase the sensitivity of nanoparticle profiling in sheathed flow.

NTA has been extensively used for counting and estimating the size of particles based on their Brownian motion in suspension. This platform has a high-resolution capability, detecting biological particles as small as 30 nm; however, measurements of larger particles (>1 µm) tend to be less accurate due to their slower movement [25,30]. Aside from size and concentration, multiple additional parameters can be analyzed, such as zeta potential, volume, surface area, light intensity, and aspect ratio of particles, providing a multifaceted biophysical assessment of polydisperse samples. More recently, NTA platforms developed optimized fluorescence modes (F-NTA), which permit a phenotypic characterization of analyzed particles within a sample [4,31,32]. Fluorophore stability and intensity pose as crucial factors for precise measurement of smaller particles, while the sensitivity required for the reliable capture of such signals renders instruments more susceptible to background noise and contaminants.

All sEVs are structurally similar. They are enclosed by a lipidic membrane, within a well-defined size range and carry different types of biomolecules found in respective donor cells. Cargo loading and release, though not fully elucidated yet, are actively regulated mechanisms that form the unique identity of each sEV and are thus responsible for the wide heterogeneity between vesicle subpopulations [33,34,35,36,37]. Identification and characterization of true sEVs requires a biochemical analysis of their content, often relying on the presence or absence of surface proteins on the lipidic membrane (i.e., classical tetraspanins CD9, CD63, and CD81). Subpopulations containing internal markers of interest can also be identified with specific membrane-permeable dyes.

Recently, several strategies for EV labeling have been proposed. These often consist of adapting staining protocols of fluorescent dyes intended to be applied in cells [20]. Dyes used for EV labeling were selected based on their specificity for different EV components such as proteins, lipids, or nucleic acids. Moreover, we applied fluorescently labeled antibodies to probe for specific outer membrane molecules. sEVs were characterized on nFCM, a dedicated high-resolution nanoflow cytometry platform that combines single-particle fluorescence detection with respective scattered light, suitable for biological particles down to 40 nm. To understand whether optimized staining protocols for purified EVs or cell-conditioned media (CCM) could be transversally applied between different platforms, we further tested them in F-NTA. Finally, we underline some of the main hurdles to single sEV discrimination, which are often related to potential co-isolated contaminants, such as large protein complexes, soluble proteins, or cell culture media components.

## 2. Results

### 2.1. EV Labeling with Membrane and Cytoplasmic Dyes

**Figure 1:**
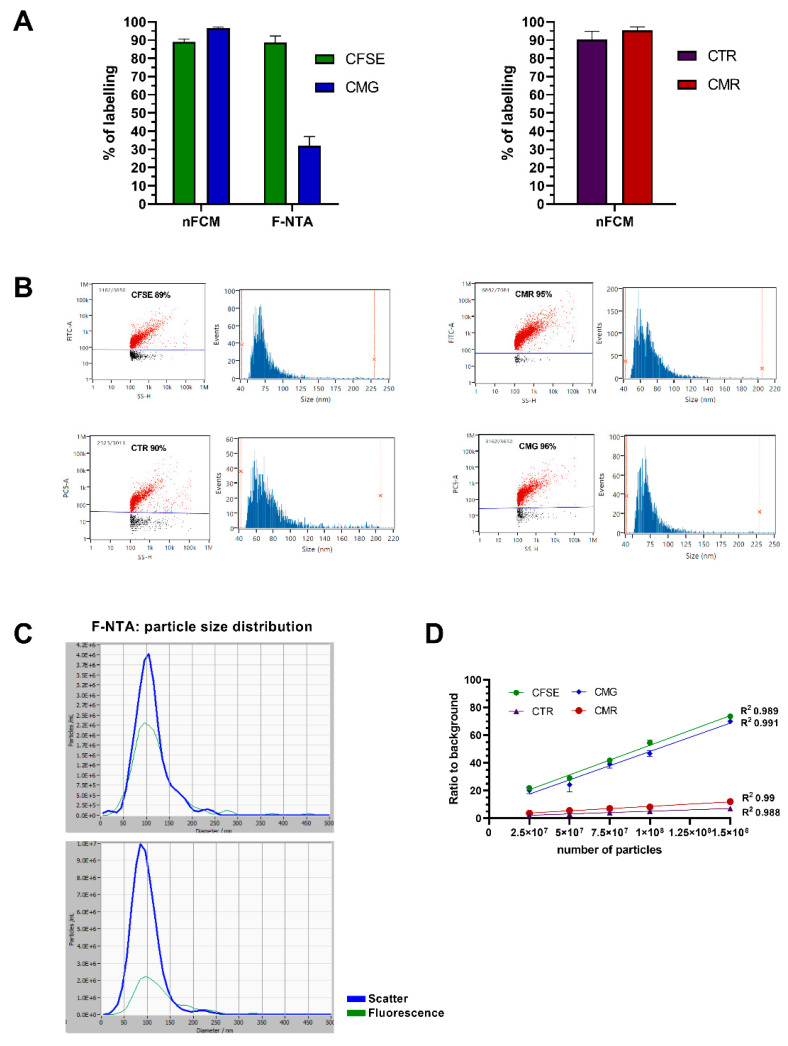
Fluorescence measurements on nFCM, F-NTA and plate reader after sEV staining with membrane and cytoplasmic dyes. (**A**) HT29 sEVs were stained with membrane-specific (CMG or CMR) or cytoplasmic dyes (CFSE or CTR) and analyzed on nFCM and F-NTA to evaluate labeling %. Data is presented as mean ± SEM of at least three independent experiments (except for nFCM-CMG and CMR, and F-NTA-CFSE, which were repeated twice). (**B**) Representative nFCM dot-plots and PSD histograms, and (**C**) histograms obtained after analyzing EVs by F-NTA in scatter and fluorescence mode. (**D**) Bulk fluorescence intensity of a dilution series of stained HT29 EVs was measured by plate reader. Signal over background (PBS) ratios are represented and trendlines drawn for the assessment of correlations. Additional data from procedural controls is provided in Supplementary Figure S1.

Different staining approaches were evaluated in this study. One consisted of staining the lipidic membrane constituents of EVs using amphipathic molecules, such as the CellMask™ (CM) Plasma Membrane Stains, CMG and CMR, which emit green and red fluorescence, respectively. Secondly, we exploited the properties of CellTrace™ cell proliferation dyes, in this case, carboxyfluorescein succinimidyl ester (CFSE) and CellTrace™ Red (CTR). These dyes differ only in the wavelength of emitted fluorescence (CFSE: green; CTR: Red), but their mechanism of action is identical. Briefly, dye molecules easily penetrate the lipidic bilayer of EVs, becoming activated by an enzymatic cleavage and covalently bound to proteins present in the EV lumen. This process effectively traps dye molecules inside every single EV, generating a stable fluorescent signal [38,39]. Over 90% of the particles in the HT29 sEV samples were stained by CMG, CMR, CFSE and CTR and detected on nFCM (Figure 1A). Larger sEVs displayed notably higher fluorescence intensities on dot plots, which was likely elicited by the incorporation of more dye molecules (Figure 1B). Staining protocols were also tested on F-NTA, with the scope of validating them on a state-of-the-art orthogonal platform. Staining performance with CFSE was ~88% on F-NTA, comparable to the one detected on nFCM (Figure 1A), although a higher concentration of dye was required to reach a staining plateau (50 µM in F-NTA, with respect to 10 µM used in nFCM measurements). On the other hand, CMG displayed a consistently lower staining efficiency, with a maximum of ~32% obtained at the concentration of 20× CMG (Figure 1A). Particle size distribution (PSD) histograms demonstrated a prevalent detection of larger particles on F-NTA, as they harbored more dye (Figure 1C—CMG). Bulk fluorescence signal-to-background ratios measured on a microplate reader varied linearly with the number of stained particles for all dyes, although the highest sensitivity was obtained with both green dyes, CFSE and CMG (Figure 1D).

To control dye specificity, besides the compulsory use of blanks and unstained controls (Supplementary Figure S1), a protein called thyroglobulin was included in this study. Due to its large size, single events could be picked up in the side scatter channel. Hardly any thyroglobulin particles were stained by CMG, however, after CFSE staining the number of detected fluorescent events in F-NTA surpassed the scattered ones by orders of magnitude, even after 300 kDa ultrafiltration (UF) washing (Supplementary Figure S5). In nFCM, CFSE-labelled thyroglobulin particles were also detected (data not shown). A comparable degree of dye activation was observed in F-NTA with CFSE-stained BSA controls, while UF successfully removed all fluorescent events (Supplementary Figure S5).

### 2.2. Characterization of sEV Subpopulations Using Single Fluorophore Tetraspanin Labeling Strategies

**Figure 2:**
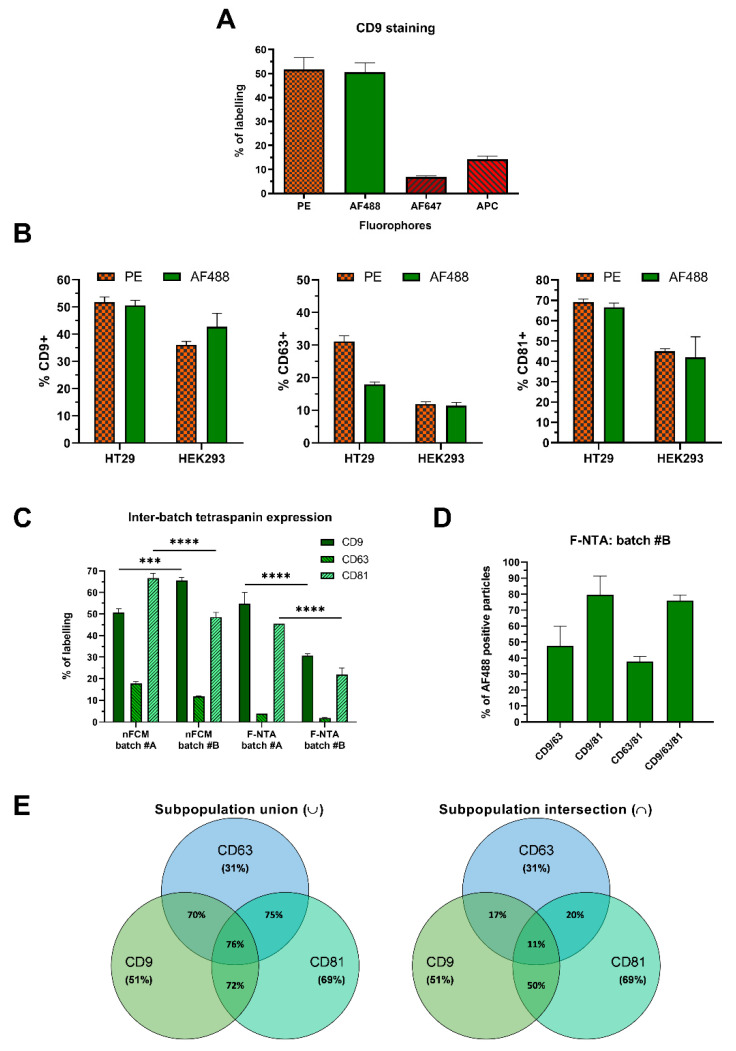
Characterization of purified sEV subpopulations based on their surface markers. (**A**) Antibody staining efficiency was evaluated by nFCM using anti-CD9 antibodies conjugated with different fluorophores. (**B**) HT29 and HEK293 sEVs were stained with anti-tetraspanin (CD9, CD63, and CD81) antibodies, conjugated with either PE or AF488, and analyzed on nFCM (*n* ≥ 3; mean ± SEM). (**C**) Cross-platform and inter-batch variability was assessed by single-staining HT29 sEV (batches #A and #B) with anti-tetraspanin AF488 antibodies (*n* = 3; mean ± SEM). Differences in tetraspanin expression between nFCM and F-NTA, and batches #A and #B, were assessed using two-way ANOVA with Tukey’s test for multiple comparisons (alpha = 0.05, *p* = 0.1234 (ns), 0.0002 (***), <0.0001 (****)). Detailed results of the statistical analysis are provided as supplementary material. (**D**) To evaluate single, as well as co-expressing, events, HT29 sEVs were stained with a mix of 2 and 3 different anti-tetraspanin AF488 antibodies and analyzed on F-NTA (*n* = 3; mean ± SEM), or on (**E**) nFCM using PE-conjugated antibodies (*n* = 3; mean ± SEM). HT29 sEV subpopulations expressing either 1, 2, or 3 markers are represented in the Venn diagram on the left. The Venn diagram on the right refers to the same HT29 sEVs, however it depicts subpopulations co-expressing both 2 or 3 markers simultaneously. Additional data from procedural controls, as well as the F-NTA PSD histograms, are provided in Supplementary Figure S1.

To identify the fluorophores conferring maximum sensitivity for sEV detection on nFCM, we compared the staining of HT29-derived sEVs obtained with anti-CD9 primary antibodies conjugated to PE, AF488, AF647, and APC. Maximal CD9 staining was achieved with PE and AF488, as both allowed detection of similar percentages of the CD9 subpopulation (close to 50%). The red fluorophores, AF647 and APC, did not stain CD9+ sEVs to a comparable degree (Figure 2A). Henceforth, in nFCM experiments we opted for PE and AF488-conjugated antibodies, having PE as reference fluorophore due to its higher brightness. Despite its extreme brightness, the high photobleaching rate of PE renders it unsuitable for the F-NTA platform, where precise measurements rely on signal acquisition for longer time periods. Therefore, AF488 was chosen for sEV phenotyping on F-NTA, since it is a more stable fluorophore.

To evaluate whether the chosen fluorophores indeed performed equally well, HT29 and HEK293 sEVs were stained with PE or AF488-conjugated anti-CD9, -CD81 and -CD63 primary antibodies, followed by nFCM analysis (Figure 2B). In HT29 sEVs, CD9 was detected at 51% (PE)/50% (AF488), CD63 at 31% (PE)/18% (AF488) and CD81 at 69% (PE)/66% (AF488). For HEK293 sEVs, detected CD9 events accounted for 36% (PE)/42% (AF488), CD63 for 12% (PE)/11% (AF488) and CD81 for 45% (PE)/42% (AF488) (Figure 2B). Both fluorophores generally allowed for the detection of similar proportions of EV subpopulations, with a major discrepancy observed only for CD63 detection in HT29 sEVs. The latter may be due to the fact that different anti-CD63 antibody clones labeled with two fluorophores were available and used in this study. To assess antibody specificity, in addition to the blank reactions, where sEVs were absent (Supplementary Figure S1), thyroglobulin was also stained as a negative control, since it is a protein particle and does not carry tetraspanin epitopes. All anti-tetraspanin antibodies caused negligible labeling, especially PE-tagged ones. CD63-AF488 revealed the highest degree of unspecific staining (Supplementary Figure S5).

Tetraspanin expression in EVs is known to vary across cell types, as well as across EV batches. When two independently harvested and purified batches of HT29 sEVs (batch #A and batch #B) were compared on both nFCM and F-NTA, we identified variations in tetraspanin expression (Figure 2C). Purified sEVs, from two different batches of HT29 CCM were stained with AF488-labelled antibodies and showed a significantly different expression level of CD9 (50.6% vs. 65.7%) and CD81 (66.6% vs. 48.7%) in nFCM, while for CD63 (17.9% vs. 11.9%) difference was not statistically significant (two-way ANOVA, Tukey’s multiple comparisons test, alpha = 0.05). Staining and analysis of batch #B sEVs in F-NTA resulted in 30.7%, 22% and 1.9% for CD9, CD81 and CD63, respectively, substantially lower percentages compared to batch #A (CD9 = 54.9%, CD81 = 45.5%; CD63 = 3.9%). Although the expression of each single tetraspanin significantly differed between batches (particularly CD9 and CD81), in F-NTA their relative trend of expression was maintained: CD9 >> CD81 >> CD63 (F-NTA batch #A and batch #B—Figure 2C). Instead, in nFCM the relative expression of tetraspanins slightly differed between the batches (batch #A CD81 >> CD9; batch #B CD9 >> CD81). From the perspective of inter-platform comparison, tetraspanin expression was significantly different across the two platforms, except for CD9 in batch #A (two-way ANOVA, Tukey’s multiple comparisons test, alpha = 0.05; detailed statistical analysis in supplementary material). A consistent trend of relative expression between platforms was obtained only in batch #B (CD9 >> CD81 >> CD63).

To analyze the single and concomitant expression of these tetraspanins, HT29 sEVs were stained with double (CD9 + CD63; CD9 + CD81; CD81 + CD63) and triple (CD9 + CD81 + CD63) antibody combinations, conjugated with PE for nFCM and AF488 for F-NTA. Nearly 76% of all particles observed on nFCM contained either CD9, CD81, CD63, or a combination of each, thereof (Figure 2E, left). The percentage of events detected upon double or triple antibody staining were not purely cumulative, which is consistent with the expectation that each sEV may express one, two, or even three tetraspanins, concomitantly. The overall portion of sEVs positive for all tetraspanins could be reproduced on F-NTA, with 76% of the particles stained by a triple antibody mix (Figure 2D). The proportion of double-positive sEVs detected on F-NTA, showed a more prominent increase when compared to previously measured single staining events, with 47.6% of particles stained with a mix of anti-CD9/anti-CD63, 79.4% with anti-CD9/anti-CD81, and 37.8% with anti-CD63/anti-CD81. Even though the signal was much higher than the sum of individually stained reactions (e.g., CD9/63 > CD9 + CD63), the patterns of expression maintained the trend expected from individual tetraspanin expression—CD9/81 >> CD9/63 >> CD63/81.

After double and triple antibody staining and understanding the expression of each single tetraspanin, sEV subpopulations co-expressing these markers could be calculated through a Venn diagram intersection analysis. Since on nFCM 70% of events co-stained with anti-CD9/anti-CD63, 72% with anti-CD9/anti-CD81 and 75% with anti-CD63/anti-CD81, we could estimate that 17% of all HT29 sEVs co-expressed CD9 and CD63, 50% co-expressed CD9 and CD81, around 20% co-expressed CD63 and CD81, and only 11% expressed all three tetraspanins simultaneously (Figure 2E). Interestingly, we noticed that CD9+ accounted for 51% and that CD9 + CD81+ for 50% of total events, which meant that all CD9+ events expressed also CD81, in our HT29 sEV samples (Figure 2E, right).

### 2.3. SEC Purification and Ultrafiltration Do Not Change the Overall Composition of sEV Subpopulations

**Figure 3:**
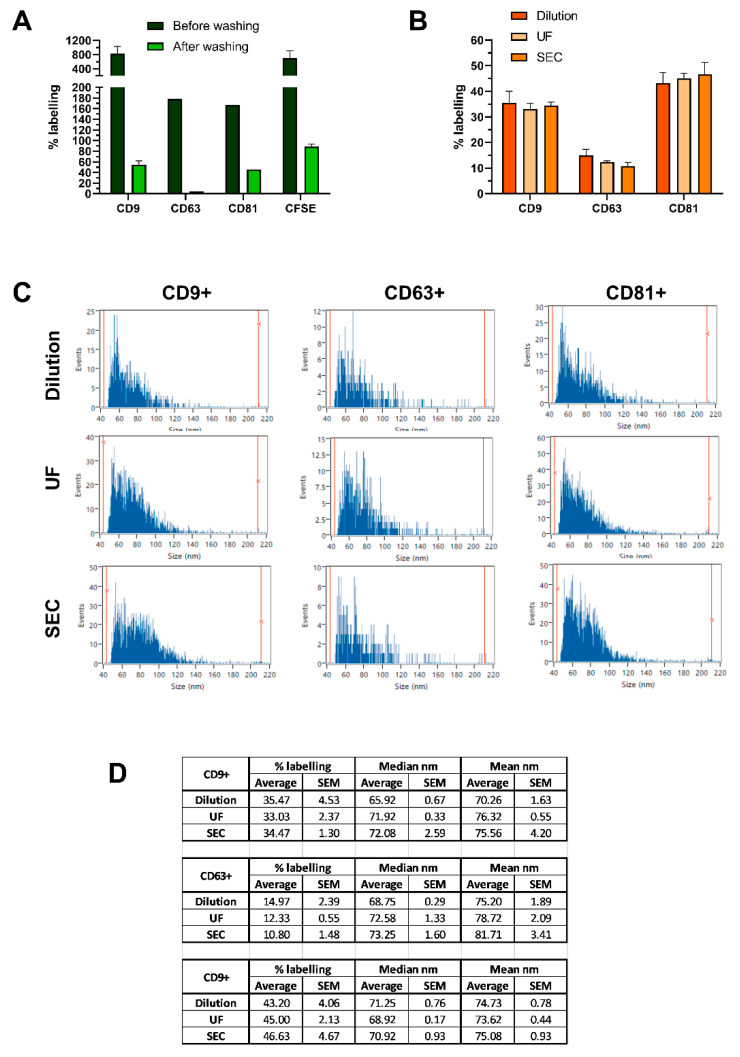
Suitability of SEC and UF as methods for clearing dyes in excess after sEV fluorescent labeling and their effect on subpopulation ratios. (**A**) Comparison of labeling % for stained HT29 sEVs before and after the removal of excess dye. 1 × 10^9^ or 5.5 × 10^9^ sEVs were incubated with antibodies (aCD9 1:12.5; aCD63 1:12.5; aCD81 1:25) or CFSE (50 µM), respectively, and measured by F-NTA. To remove the excess dye, UF washing strategy was applied, followed by F-NTA detection. (**B**) To compare different strategies for the removal of fluorescent antibodies in excess, labeling %, (**C**) PSD histograms, and (**D**) median and mean particle diameter were assessed for CD9+, CD63+ and CD81+ HEK293 sEVs, on nFCM. 2 × 10^9^ sEVs, at a concentration of 10^8^ particles/µL, were incubated with PE-labelled antibodies (aCD9 1:500; aCD63 1:25; aCD81 1:500) and unbound antibodies were removed by SEC or UF. Sample dilution (500–1000-fold) served as staining reference. Data is presented as mean ± SEM of at least three independent experiments. F-NTA PSD histograms of samples analyzed before and after washing are provided in Supplementary Figure S2.

Optimized sEV staining protocols included a filtering step for buffer exchange, which was able to retain sEVs and to remove dyes and antibodies in excess. After testing several filtration devices, Nanosep 300 kDa ultra-filters and SEC were chosen for this purpose. One major concern was that background fluorescence could mask the mild fluorescent signal derived from fluorescently labeled nanoparticles and impede the acquisition of true positive events, resulting in skewed measurements. Indeed, free dye and antibodies impinged on sEV analysis, as clearly demonstrated on F-NTA where the fluorescence background led to overestimations of labeling efficiency (Figure 3A). PSDs of fluorescently labeled sEVs were also affected, as the size distributions shifted to the left, revealing a peak below 50 nm (Supplementary Figure S2). Similarly, on nFCM the excess of free fluorophores in solution led to increased thresholds in fluorescence channels. Therefore, filtering proved to be a critical step for the reliable detection of labeled vesicles, as even traces of free dyes and/or antibodies generated artifacts and overwhelming fluorescence noise on both instruments.

Nonetheless, we questioned whether these buffer exchange methods could inadvertently select specific sEV subpopulations and thus result in biased analysis. To this end, HEK293 sEVs (10^8^ particles/µL) were stained with PE-conjugated anti-CD9, -CD63, or -CD81 and detected subpopulations compared after sample over-dilution, SEC and UF at nFCM. Sample over-dilution consisted of diluting a complete staining reaction in PBS until the concentration of the unbound fluorescent antibody was low enough to avoid background fluorescence, while sEV concentration was maintained at the optimal range for measurement. Since the over-dilution approach is inherently unbiased, it functioned as reference staining. Regardless of the method applied, we observed no significant difference between tetraspanin expression levels (Figure 3B), suggesting that neither SEC nor UF alters the composition of sEV subpopulations. This was also supported by the mean and median values of sample PSDs (Figure 3C,D).

### 2.4. Identification and Quantification of sEV Populations upon RNA Staining

**Figure 4:**
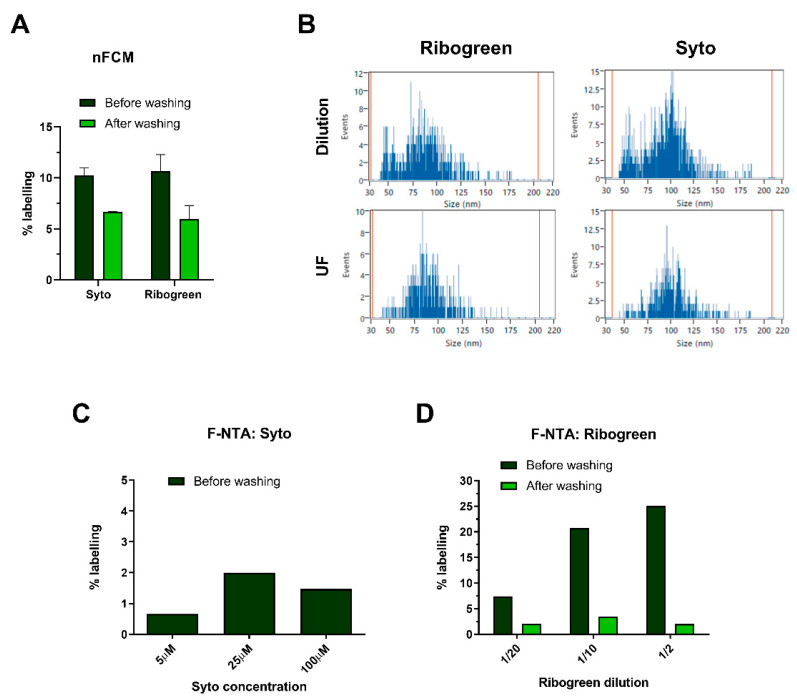
Identification and quantification of sEV populations based on their RNA content. (**A**) HT29 sEVs were stained with the RNA dyes Syto (25 µM) and RiboGreen (1:50). After incubation, staining reactions were washed by UF and fluorescent particles subsequently measured, with respective (**B**) PSDs analyzed on nFCM. (**C**) HT29 sEVs were incubated with Syto at increasing concentrations and the % of positive events was read on F-NTA, without applying further washing steps. (**D**) The impact of UF washing was evaluated on labeled HT29 sEVs, at increasing concentrations of RiboGreen. Fluorescent subpopulations were detected using F-NTA. Staining for nFCM was performed in three independent experiments (*n* = 3; mean ± SEM), while for F-NTA, representative results of titration experiments are shown for both dyes. Additional data from procedural controls, as well as the F-NTA PSD histograms of analyzed samples, are provided in Supplementary Figure S3.

Aiming to further characterize HT29 sEV subpopulations, we evaluated their nucleic acid content with membrane-permeable dyes, specific for RNA-SYTO™ RNASelect™ and Quant-iT™ RiboGreen™. Since both dyes only exhibit their full brightness upon binding to RNA, the background signal generated by unbound dye molecules should be drastically reduced compared to PE or AF488. Such reasoning prompted us to explore the suitability of the sample over-dilution approach in this context. After staining reaction over-dilution, fluorescent subpopulations detected by nFCM averaged around 10.2% and 10.6% for Syto and RiboGreen, respectively (Figure 4A). However, UF washing caused a consistent drop in fluorescent events for both dyes, with respect to the sample over-dilution reference. Loss of fluorescent events after UF also reflected on PSDs, as a noticeable reduction in particles with smaller diameters was observed (Figure 4B).

Similar observations were made after analyzing Syto and RiboGreen-stained HT29 sEVs on F-NTA. No significant fluorescence signal was detected with Syto (<2%), even at the highest concentration of dye used (100 µM) (Figure 4C). Conversely, sEVs stained with different concentrations of RiboGreen reached up to 25% of labeling efficiency (Figure 4D). Notably, detected fluorescent particles showed a tendency towards larger PSDs (Supplementary Figure S3). Nevertheless, after washing the samples with UF, the fluorescent signal was completely lost (Figure 4D). Blank controls (without sEVs) showed a negligible number of fluorescent events on both instruments, indicating that free dye alone did not generate false-positive counts, either before or after washing (Supplementary Figure S3).

### 2.5. Identification and Quantification of sEVs Directly in Cell-Conditioned Media

**Figure 5:**
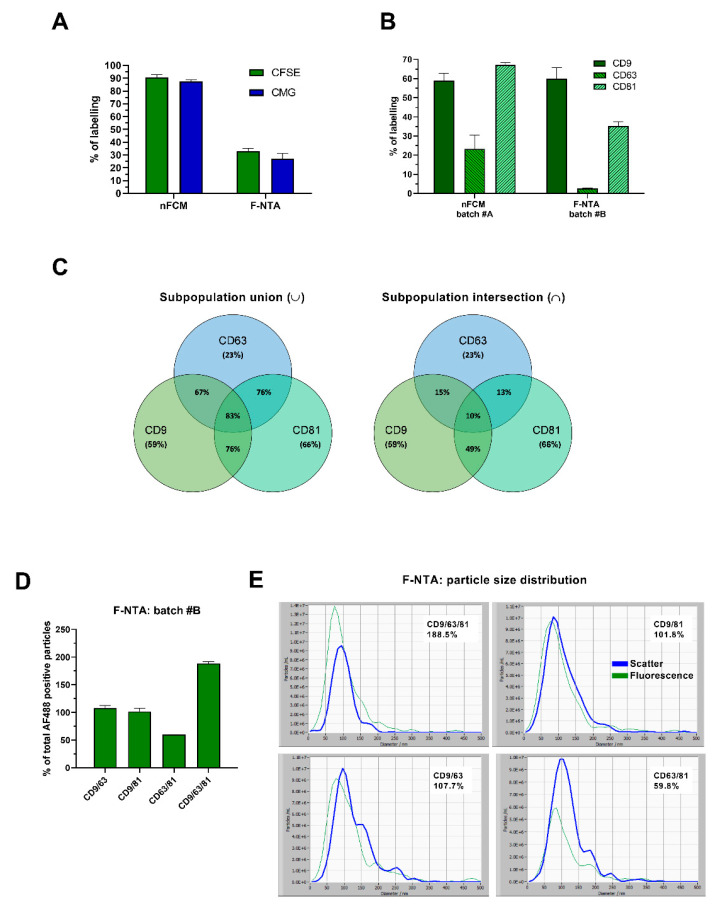
Characterization and quantification of sEVs directly from non-purified cell-conditioned media. HT29 CCM particles were stained and analyzed following the optimized protocols for sEV labeling with CMG, CFSE or fluorescently labeled anti-tetraspanin antibodies. (**A**) Staining efficiencies of CMG-, CFSE-, and (**B**) single antibody-labeling were assessed on both platforms. (**C**) Co-expressing markers were also evaluated on HT29 CCM by applying a mix of 2 and 3 anti-tetraspanin, PE-conjugated antibodies for nFCM measurements. The Venn diagram on the left depicts subpopulations that carried 1, 2, or 3 tetraspanins, while the one on the right represents sEV subpopulations which expressed 2 or 3 markers simultaneously. (**D**) HT29 CCM staining employing mixes of 2 and 3 AF488-labelled anti-tetraspanin antibodies was carried out for F-NTA, and staining efficiencies, as well as the (**E**) PSDs in scatter and fluorescence modes were assessed. Data represents triplicate experimental points (*n* = 3; mean ± SEM), except for (**C**) which was performed in two independent experiments (*n* = 2; mean ± SEM). F-NTA PSD histograms of CCM samples after CFSE-, CMG- and single antibody-labelling, are provided in Supplementary Figure S4.

Purified EVs are ideal for a single-particle characterization. However, purification can be lengthy, labor-intensive, and biased if the process enriches certain EV subpopulations. To evaluate the possibility of avoiding sEV purification from CCM, while still accurately detecting sEV subpopulations, previously optimized staining protocols were directly applied in HT29 CCM and particles were measured by nFCM and F-NTA. As shown in Figure 5A, CFSE and CMG labeled ~90% of events when analyzed on nFCM, well recapitulating the results obtained with purified HT29 sEVs (Figure 1A). On the contrary, CFSE and CMG staining on HT29 CCM resulted in only 33% and 27% of labeling on F-NTA, respectively (Figure 5A). Peak and overall PSD of fluorescent subpopulations leaned towards higher values, as opposed to total particles measured in a scatter mode (Supplementary Figure S4).

Direct incubation of anti-tetraspanin antibodies in HT29 CCM and subsequent nFCM analysis revealed slight variations in the tetraspanin expression profiles, with respect to those reported for purified HT29 sEVs. Percentages of positive events were 59% for CD9, 67% for CD81 and 23% for CD63 (Figure 5B). On F-NTA, percentages of staining with AF488 antibodies were 59.8% for CD9, 35.3% for CD81, and 2.6% for CD63 (Figure 5B), higher than in purified HT29 sEVs from the corresponding batch (Figure 2C, F-NTA). Nevertheless, the same trend of expression was maintained between HT29 CCM and HT29 purified sEVs (CD9 >> CD81 >> CD63). Detailed statistical analysis by two-way ANOVA, comparing variability in tetraspanin expression levels between purified (sEVs) and non-purified (CCM) samples, is provided in the supplementary material.

In line with the purified sEV experiments reported above, we evaluated the concomitant expression of the three tetraspanins in HT29 CCM, applying combinations of CD9, CD81, and CD63 antibodies, PE-conjugated for nFCM and AF488-conjugated for F-NTA. Nearly 83% of the detected particles on nFCM displayed either CD9, CD81, or CD63, slightly more than the 76% previously determined for purified sEVs from the same CCM batch (Figure 5C). Regarding tetraspanin co-expression on nFCM, 15% of events carried both CD9 and CD63, almost 50% displayed both CD9 and CD81 and 13% were positive for both CD63 and CD81, while 10% resulted positive for all the three markers. The profile of tetraspanin expression was identical to the one obtained in purified sEVs, evidencing a maximum fluctuation of only 7% (Figure 2E and Figure 5C).

AF488-conjugated antibody combinations did not reproduce the same values on CCM, as observed for purified EVs on F-NTA. Double and triple staining reactions evidenced unrealistic fluorescent event numbers, yielding over 100% of events detected in scatter mode. The combination of multiple antibodies in a complex, non-purified biofluid likely resulted in poor removal of unbound antibodies in excess (Figure 5D). Background fluorescence hampered correct analysis, as shown by the shift in PSD histograms between scatter and fluorescence mode (Figure 5E).

### 2.6. Characterization of sEV Subpopulations Using Multicolor Fluorescence Labeling Strategies

**Figure 6:**
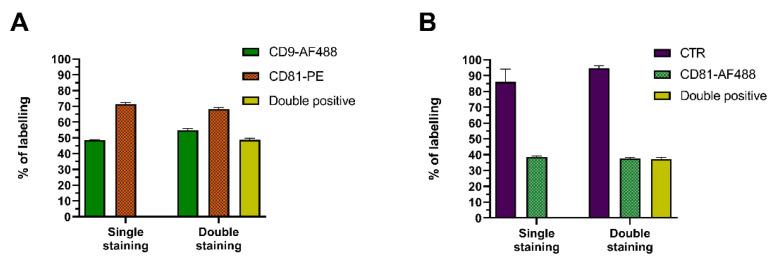
Multiplex fluorescence analysis for enhanced sEV identification and characterization. (**A**) Purified HT29 sEVs were labeled with anti-CD9-AF488 and anti-CD81-PE either individually (single staining) or in combination (double staining). (**B**) HT29 sEVs were stained with CTR and anti-CD81-AF488 either individually or in combination. Results are shown as % of labeled particles detected on nFCM, in two fluorescence channels (*n* = 3; mean ± SEM).

Depending on the complexity of biological samples, a wide range of contaminants can co-purify with sEVs, adding up to the already high heterogeneity of subpopulations. Consequently, it is crucial to pinpoint and discriminate true sEVs from confounding particles, whilst extracting additional information about their nature and/or contents. This prompted us to attempt multiple labeling strategies, combining different dyes and antibodies. Firstly, purified HT29 sEVs were stained with CD9-AF488 and CD81-PE alone, to individually determine the expression of each marker. Then, both antibodies were combined, and the staining efficiency was compared. CD9-AF488 alone stained 50% of particles and in combination with CD81-PE, this number slightly increased to 55%. The labeling efficiency obtained with CD81-PE was 70% in single staining and 68% after incubation together with CD9-AF499 (Figure 6A). As for the double-positive CD9+/CD81+ subpopulation, 48% of the particles displayed a double fluorescent signal, which perfectly matched the co-expression level of CD9 and CD81, previously identified in HT29 sEVs using a combination of strictly PE antibodies (Figure 2E and Figure 6A). In line with the goal of further distinguish the nature of antibody-labeled particles, we attempted to optimize a double staining protocol applying CTR and CD81-AF488. The labeling obtained for each individual dye matched well with single stain controls. It was observed that the entire CD81+ subpopulation could be simultaneously stained with CTR, thereby supporting the presence of true sEVs (Figure 6B). Surprisingly, HT29 sEVs expressed nearly 40% of CD81 in this experiment, whereas in previous ones it was detected at 65–70% (Figure 2B).

## 3. Discussion

In the present study, we compared single nanovesicle profiling platforms, aided by fluorescent labeling with different dyes and antibodies. To this end, we evaluated the capabilities of two platforms, the recent nFCM and the more established NTA, which has been recently upgraded for compatibility with fluorescence measurements—F-NTA. Although both instruments perform single-particle analysis, there are crucial differences in their hardware components and mode of operation. nFCM gathers scattered light to estimate particle size, while F-NTA calculates their hydrodynamic diameter by tracking particle diffusion motion.

The accuracy of nFCM particle size estimations relies on calibrating the instrument using silica beads with refractive properties similar to those of sEVs. The resulting calibration curves are in accordance with the Rayleigh scattering theory as they fit the expected model for the light scattering of particles smaller than the wavelength of incident light [28]. However, the refractive index (RI) of silica beads (1.46) does not exactly match the RI of EVs (1.36–1.4), and given the heterogeneity of EV sizes and biomolecular scaffolding, even greater differences in refractive properties between subpopulations of particles could arise [40,41]. To account for these limitations, size estimation in nFCM has implemented Mie scattering theory calculations, which adjust calibration curves to minimize any potential errors stemming from differences between size standards and EVs [42,43,44].

On the other hand, NTA requires a longer acquisition time window to determine particle size and the analysis of polydisperse samples imposes protocol readjustments to encompass a wider range of sizes [30]. Additionally, accurate size estimation based on Brownian motion becomes challenging with larger particles due to their slower diffusion, which could be affected by the EV surface composition, medium viscosity, and temperature [5,45,46,47]. The strength of NTA lies in the fact that it is a well-established method and does not rely on RI, which provides great flexibility for measuring nanoparticles of different compositions without the need for reference material in each analysis. However, when it comes to fluorescent labeling and detection, NTA poses certain limitations (bright and stable fluorophores; longer signal acquisition time) and requires further development and optimization. Furthermore, avalanche photodiodes (APD) in nFCM might allow for higher resolving power and better signal detection, especially in fluorescence mode, when compared to CMOS camera sensors [48].

In this study, F-NTA measurements required more washing cycles to completely eliminate background fluorescence, than nFCM. Stronger laser power (15 mW in nFCM vs. 40 mW in F-NTA), the fact that F-NTA acquires fluorescent signal from a stationary liquid in a cell for a longer fraction of time, compared to a fast detection in continuous flow on nFCM, and that nFCM applies SSC-triggered measurements of fluorescence, might be some of the reasons for a higher susceptibility to background noise on F-NTA. This was especially the case when analyzing more complex biofluids, such as CCM, drawing attention towards limited EV analysis in non-purified matrices.

Despite the need for more washing cycles, the analysis of purified sEVs could be carried out on both instruments. Cytoplasmic dyes, particularly CFSE, performed comparably well on both instruments, representing an optimal balance between fluorescence intensity and photostability. CFSE and cell-trace dyes theoretically require enzymatic cleavage by esterases to covalently bind to intraluminal proteins and become fluorescent. Several studies employing various nanoparticle profiling platforms have applied CFSE as a way to selectively stain EVs [20,21,22,23,24,49,50]. Nonetheless, CFSE and cell-trace dyes should be used with caution, since in our hands they became activated independently of intraluminal esterases. The presence of non-vesicular proteins and potential contamination with soluble esterases may cause CFSE activation—as evidenced by the staining of thyroglobulin particles and by the significant number of fluorescent artifacts in BSA controls reported in this work. Cell membrane dyes (CMG, CMR) did not exert the same extent of background fluorescence; however, their staining efficiency was sub-optimally detected in F-NTA experiments.

For ideal single nanoparticle profiling, it was important to choose widely available, photostable, and high brightness fluorophores. On nFCM, AF488 and PE performed equally well and surprisingly, allowed for increased sEV staining efficiencies over the red fluorophores AF647 and APC, even though AF488 theoretically should have the lowest brightness (extinction coefficient x quantum yield) out of them all (see the Supplementary Table S1). Generally, red fluorophores may also be more prone to self-quenching, consequently diminishing their quantum yield [51]; therefore, it would be relevant to address the properties of such dyes within the scope of single nanoparticle analysis. For F-NTA, AF488 provided the optimal balance between stability and brightness. Despite being one of the brightest commercially available dyes, PE was omitted from F-NTA measurements due to its fast bleaching, which could result in the underestimation of truly stained particles.

The fact that total particle counts (scatter mode) on F-NTA were nearly 4–5 times higher than they were on nFCM might be explained by differences in laser power, as the strength of incident light sources and the composition of illuminated particles directly correlate with the intensity of scattered light and ultimately, with the number of detectable nanoparticles. On the other hand, CMOS sensors might not be as sensitive as APDs, which could lead to poorer detection of faintly expressed epitopes on the surface of sEVs, limiting the number of fluorophores associated per fluorescent event. This could help to explain the significantly lower labeling percentages detected with F-NTA during antibody staining experiments. Such reasoning is further corroborated by double and triple antibody staining experiments, where the number of fluorescent events was higher than the sum obtained after each single staining (Figure 2C,D). EVs displaying few CD9, CD63 or CD81 epitopes on their surface would remain undetectable on F-NTA until multiple tetraspanins are labeled (Supplementary Figure S6). Nevertheless, F-NTA still provided consistent results between batches, revealing a trend in tetraspanin expression that was comparable in the case of single (CD9 >> CD81 >> CD63) as well as multiple antibody reactions (CD9/CD81 >> CD9/CD63 >> CD63/CD81).

These limitations did not seem to occur in nFCM, however, CD63-PE and CD63-AF488 resulted in 31% and 18% of staining on HT29 sEVs, respectively (Figure 2B). The fact that suppliers and clones were different between PE and AF488 antibodies could explain this discrepancy, though it was noticed only for CD63 and on HT29 samples; CD63 staining efficiencies using PE or AF488-conjugated antibodies were equal on HEK293. Notably, CD63 protein is reported to have different isoforms deriving from different splicing variants or post-translational modifications that may have functional or morphological implications and affect their partnering with other membrane molecules [52]. Therefore, the potential specificity of certain Ab clones for cell types or conditions must be better understood. Another possibility aligns with the phenomenon described just above, since CD63 was the least abundant tetraspanin in this study, AF488 staining may miss events carrying very few epitopes. Generally, nFCM was able to better discern fluorescently labeled EVs and also enabled the characterization of multiple surface markers through a single-color fluorescent analysis, which is extremely valuable, especially in dedicated high-resolution platforms that are limited to a few channels for fluorescence detection. Therefore, we can argue that nFCM is more sensitive and consistent for fluorescent measurements.

For applications where sEV purification is not feasible, or minimal sample processing is a concern, we questioned whether our protocols for sEV fluorescent labeling could be applied directly in more complex biological samples such as CCM. Results between purified sEVs and CCM sEVs were surprisingly similar on nFCM. On the other hand, F-NTA was more promiscuous—lower fluorescent signal with CFSE in CCM could mean the presence of many non-EV particles, however, antibody staining gained percentages that were significantly higher than those obtained with purified sEVs. Behind this contradiction may be a reduced antibody washing efficiency, attributed to the richness of CCM. Double and triple antibody staining experiments further supported this hypothesis, where fluorescence background was even higher, leading to the conclusion that for background-free F-NTA measurements, purified material is preferred, or alternative washing procedures should be used instead of UF. It should be noted that the efficiency of UF washing was reduced when presented with CCM samples and multiple-antibody staining reactions.

We also assessed the feasibility of a sample over-dilution approach as an alternative staining protocol that avoids further processing for dye removal. Staining reactions had been previously optimized, maintaining a fixed range of sEVs (10^8^–10^9^), while titrating both dyes and antibodies to determine their optimal concentrations (at which a staining plateau was reached). For dyes, the dilution approach was not feasible for either of the instruments, as at the optimal dilution for the sample measurement, background fluorescence levels were still massive. On F-NTA, this problem also persisted when the over-dilution was applied to anti-tetraspanin antibody staining, confirming the need for a washing step after optimized staining reactions. Conversely, this protocol could be applied in nFCM, though it required an elevated concentration of purified sEV input (up to 10^8^ per microliter). This allowed for staining reactions in lower volumes, minimizing the amount of antibody while maintaining optimized concentrations. Samples could be directly analyzed after a 500–1000-fold dilution, without any loss in sEV staining efficiency, with respect to UF or SEC.

The sample dilution-based protocol also featured as a reference method to evaluate possible biases introduced by UF or SEC, employed in this study for sEV buffer exchange and as a benchmark separation method for EV isolation. We confirmed that regardless of the dye removal method, there were no significant differences between CD9, CD81, or CD63 subpopulations. In this way, we demonstrated that neither SEC nor UF alters the composition of sEV subpopulations (Figure 3B). Similar mean and median size values also supported this claim (Figure 3C,D). Throughout the study, we favored the use of 300 kDa UF devices, as it is a more practical method than SEC and also confers higher sample processing throughput.

Overall comparison of washed and unwashed sEV samples proved to be useful in the early assessment of the efficacy and specificity of sEV staining protocols, including nucleic acid-specific dyes. The use of RNA-specific dyes for EV characterization has been reported in earlier works [53,54,55]. In this study, both SYTO™ RNASelect™ and Quant-iT™ RiboGreen™ revealed a low abundance of RNA-containing HT29 sEVs. As the activation of these dyes is dependent on nucleic acid binding, interference caused by background fluorescence signal seemed unlikely, hence the sample over-dilution approach was successfully applied. The low percentage of stained events questions not only the overall amount and accessibility of EV RNA but also the brightness of hereby employed dyes.

Following the MIFlowCyt-EV guidelines [56], procedural and assay controls, as well as washing steps, were included in all the experiments. Similarly, staining reactions with RNA dyes were also subjected to UF. This reduced the percentage of fluorescent events, leading to the conclusion that RNA staining may not be as specific or stable as with other dyes and antibodies. Such a decrease in fluorescent events after UF washing raises concerns on the possible artifacts generated by dyes, as well as on the nature and strength of nucleic acid association to EVs. Given that RNA dyes can minimally bind to DNA molecules, the overall weak fluorescent signal could be justified by a low affinity binding to extravesicular DNA, which would then be lost upon UF washing. The presence and topology of nucleic acids on EVs have been the topic of discussion in previous publications [35,36,57,58,59], which might pose more critical approaches to assess the true EV nucleic acid content, location, and their usefulness as biomarkers. It is also possible that fluorescent events are not sEVs, but rather large individual ribonucleoprotein complexes [35,54,60,61]. This could be addressed through co-staining experiments with RNA-specific dyes and anti-tetraspanin antibodies. Although their blank controls show a low presence of fluorescent events, we cannot exclude the possibility that SYTO and RiboGreen may nonspecifically adsorb to EVs.

Since nFCM is equipped with two fluorescence channels, it allows for colocalization analysis. Dual fluorophore labeling proved to be a viable strategy for sEV subpopulation assessment, using either a combination of two antibodies or one antibody with another dye, as long as their emission spectra are sufficiently far apart to avoid fluorescence bleed-through between channels. The downside of this approach lies in its prolonged incubation time, which may have resulted in lower staining efficiency—HT29 sEVs expressed nearly 40% of CD81 after double-staining, whereas after single-staining CD81 was detected at 65–70% (Figure 2B and Figure 6B). In conclusion, after careful optimization of staining protocols, it is possible to combine multiple fluorophores efficiently, to enable multiparametric sEV characterization.

## 4. Materials and Methods

### 4.1. Cell Culture and sEV Isolation

Cell lines HT29 (ATCC^®^ HTB-38™, Manassas, VA, USA) and HEK293 (ATCC) were expanded in McCoy’s and DMEM growth media (Euroclone, Pero, Italy), respectively, supplemented with 10% FBS (Euroclone) and 1% pen/strep (Sigma, St. Louis, MO, USA). Cells were grown in T75 or T150 Flasks and maintained in a humid atmosphere of 5% CO_2_ and at 37 °C. Once expanded to the desired confluence (80%), cells were washed 2 times with 1× PBS and conditioned in a serum-free medium (to avoid serum-derived confounding particles) for 48–72 h. CCM was harvested and clarified by differential centrifugation at (1) 300× *g* for 10 min, (2) 1200× *g* for 20 min, and (3) 10,000× *g* for 30 min at 4 °C. Pre-cleared CCM was directly used for experiments, further processed for EV purification and isolation, or stored at −80 °C.

For sEV isolation, CCM was concentrated using an Amicon Stirred Cell ultrafiltration unit (Ultracel 100 kDa Ultrafiltration Discs, Merck Millipore, Burlington, MA, USA). A maximum volume of 500 mL of CCM was concentrated down to 10 mL for each isolation. Concentrated CCM was fractionated in size-exclusion chromatography (SEC) columns (Sepharose CL-4B bed volume 70 mL, GE Healthcare, Chicago, IL, USA), pre-equilibrated with 1 × 0.22 µm filtered PBS. Fractions of 1 mL were collected and EV-containing fractions 16 to 40 were pooled (total volume ≈ 25 mL). SEC-purified sEVs were concentrated down to 0.5–1 mL by 100 kDa ultrafiltration (Amicon^®^ Ultra-15 Centrifugal Filter Unit, Merck Millipore).

### 4.2. Staining Protocols

#### 4.2.1. Staining with Cytoplasmic, Membrane, or RNA-Specific Dyes

To ensure the optimal working concentrations of dye for our platforms, incremental concentrations were tested on a fixed number of particles (2 × 10^9^). Dye concentrations at which the percentage of stained events reached a plateau were henceforth applied. After determining particle concentration (10^7^ to 10^8^ particles/µL), between 5 × 10^8^ to 2 × 10^9^ particles (purified sEVs or CCM) were loaded in each staining reaction with CellTrace™ CFSE (CFSE; 10 µM; Thermo Fisher Scientific, Waltham, MA, USA), CellTrace™ Far Red (CTR; 15 µM; Thermo Fisher Scientific), CellMask Green (CMG 20×; Thermo Fisher Scientific) and CellMask Red (CMR 20×; Thermo Fisher Scientific), in filtered PBS. Reactions were incubated for 1.5 h at 37 °C under shaking, protected from light. After incubation, reaction volumes were brought up to 500 µL with filtered PBS and loaded into SEC columns (Sepharose CL-4B bed volume 10 mL, GE Healthcare) for the removal of unbound dye in excess. Fractions of 500 µL were collected and the EV-containing fractions (7, 8 and 9) were pooled, resulting in a total of 1.5 mL of stained sEV samples, which were immediately analyzed. SYTO™ RNASelect™ (Syto; Thermo Fisher Scientific) and Quant-iT™ RiboGreen^®^ (RiboGreen; Thermo Fisher Scientific) were added at 25 µM and diluted 1:50, respectively. For Syto and RiboGreen, samples were incubated for 1.5 h and 30 min, respectively, both at 37 °C under shaking, protected from light. Afterward, they were washed 3–4 times with PBS using ultrafiltration centrifugal devices (Nanosep^®^ 300 K, Pall Corporation, Port Washington, NY, USA; hereinafter referred to as UF) and analyzed.

As the ZetaView PMX-120 NTA instrument (Particle Metrix, Inning am Ammersee, Germany; in this text referred to as F-NTA) has only one laser (488 nm), red dyes were not used on this platform due to their longer excitation wavelengths. Furthermore, F-NTA detected 4–5.5 times more total particles than the Flow NanoAnalyzer (model U30, nanoFCM Inc., Xiamen, China; in this text referred to as nFCM), hence the particle amount and dye concentration for staining reactions were additionally optimized on this platform. For F-NTA analysis, 1 × 10^9^–5.5 × 10^9^ of particles (purified sEVs or CCM) were incubated with CFSE (50 µM) and CMG (20× concentrated) for 1.5 h at 37 °C. Afterward, the excess dye was removed by repeated washing (6–8 times) with 500 µL PBS and UF. Washed samples were measured in both scatter and fluorescence mode. Titration was also conducted for Syto (5, 25, and 100 µM per reaction) and RiboGreen (1/20, 1/10, and 1/2 dilution per reaction), samples were incubated for 1.5 h and 30 min at 37 °C, respectively, after which only RiboGreen stained samples were washed 8 times with PBS using UF. Samples were then analyzed in both scatter and fluorescence mode.

In both platforms, unstained samples and blanks (PBS and dye) were used as procedural and assay controls. The number of thyroglobulin (Merck/Sigma-Aldrich) particles was equivalent to the number of sEVs per staining reaction and the same protocols applied. Additionally, 20 µg of purified bovine serum albumin (BSA) standards (Thermo Fisher Scientific) were used in a control reaction of CFSE specificity.

#### 4.2.2. Single Fluorophore Antibody Staining

Optimal working dilutions for antibodies were determined following the same approach described above. For each staining reaction in nFCM, 2 × 10^8^–2 × 10^9^ particles (purified sEVs or CCM) were incubated with fluorescent primary antibodies for 1 h at 37 °C under shaking, protected from light. Next, PBS was added up to 500 µL, unbound antibodies were removed by 3 to 4 rounds of UF or SEC and stained samples were characterized. The following fluorescently-labeled primary antibodies were used: Phycoerythrin (PE)-conjugated mouse anti-human CD9, CD63 and CD81 (dilution 1:10 for all; Exbio, Vestec, Czech Republic), Alexa Fluor^®^ 488 (AF488)-conjugated mouse anti-human CD9, CD63 and CD81 (1:500, 1:25 and 1:500, respectively; R&D Systems, Minneapolis, MN, USA), allophycocyanin (APC)-conjugated mouse anti-human CD9 (1:10; Exbio), Alexa Fluor^®^ 647 (AF647)-conjugated mouse anti-human CD9 (1:10; Exbio). Since PE is quite susceptible to photobleaching and APC and AF647 require a 600–640 nm excitation wavelength, only AF488-conjugated anti-human CD9, CD63 and CD81 were used in F-NTA, diluted 1:12.5, 1:12.5, and 1:25, respectively. 1 × 10^9^ particles (purified sEVs or CCM) were used for the staining reaction at 37 °C, for 1.5 h. Unbound antibodies were removed by repeated washing (6–8 times) with 500 µL PBS and UF, prior to analysis.

#### 4.2.3. Multicolor Fluorescence sEV Staining

It is important to avoid the overlap between the emission spectra of dyes to be used in combination, which results in the detection of false-positive events. With that in mind, AF488 and PE were combined for dual-color fluorescent labeling experiments with antibodies. Between 2 × 10^8^–2 × 10^9^ particles were incubated with CD9-AF488 and CD81-PE at optimal working dilutions indicated in the section above, for 1 h at 37 °C under shaking, covered from light. Antibody in excess was eliminated by 3 to 4 rounds of UF or SEC and labeled sEVs directly analyzed on nFCM. To avoid interference of dyes in the specific binding of antibodies when combining both for sEV double staining, 5 × 10^8^ to 2 × 10^9^ particles were firstly labeled with CD9-AF488 for 1 h at 37 °C under shaking, followed by incubation with CTR (15 µM) for 1 h 30 min at 37 °C under shaking. The volume of double staining reactions was brought to 500 µL with filtered PBS, unbound antibodies and dyes were eliminated by SEC, and samples were examined right after. Since our F-NTA instrument is equipped with only one laser, dual-color labeling was omitted on this platform.

### 4.3. nFCM: Instrument Setup and EV Analysis

Conventional flow cytometers are not designed to characterize small nanoparticles such as sEVs and thus may provide dubious information. For this reason, nFCM, a dedicated nanoflow cytometry platform, was employed in this study. Our instrument is equipped with two lasers (488 and 638 nm), three single-photon counting modules (SPCM) and enables simultaneous detection in three independent channels. Light is first detected in the SSC channel (bandpass filter: 488/10) and then directed by two dichroic beam splitters (DicF495; DicF555) towards the green channel (bandpass filter: 525/40) and finally to the orange/red channel (bandpass filter: 580/40 or 670/30). Before each experiment, the NanoAnalyzer was aligned using polystyrene QC beads (nanoFCM Inc.). Size and concentration standard nanospheres (nanoFCM Inc.) were read directly after in order to calibrate the instrument for sEV analysis. Once nFCM was aligned and calibrated, sEV samples were diluted in filtered PBS (blank) to the optimal range for measurement (10^8^ particles/mL). Samples and blanks (200–800 events) were measured for 1 min, applying a laser power of 15 mW as excitation source, constant pressure of 1 kPa, and at an event rate between 2500 to 12,000 events/min (as recommended by manufacturers), to avoid particle swarm detection ^14,15^. Since SSC was set as the trigger channel, each particle that generated a signal above the SSC threshold was acquired as an event. For each event that also generated a signal above thresholds set in the fluorescent channels, the fluorescence intensity was registered. Thresholds were automatically set for each sample, accounting for background signal throughout the run (thresholds = average background measurements + 2× their standard deviation). Any sample with an SSC threshold 10% higher than the reference (size standard nanospheres) was excluded from the study. Similarly, strict criteria were set for the acceptance of fluorescently labeled samples, relying on unstained sEV controls as a reference for background fluorescence. Empty staining reactions (without sEVs) were performed as a control for all fluorescent reagents and measured under the same conditions as complete reactions (with sEVs).

### 4.4. F-NTA: Instrument Setup and EV Analysis

Particle Metrix ZetaView PMX-120 (software version 8.05.12 SP1) is a nanoparticle tracking analysis instrument equipped with a 488 nm laser (40 mW of power) and CMOS camera sensor which enables enumeration, physical characterization, and fluorescence measurement of particles in suspension. Measurements can be performed in liquid samples with a minimal volume of 500 µL, containing particles as low as 10^6^. PMX-120 was set up according to the manufacturer’s instructions. Cell check was performed after each start-up, followed by camera and laser alignment using 100 nm polystyrene size standard beads and optimizing profile auto-symmetry. Upon instrument setup, daily performance was conducted with the same beads in order to assess the accuracy and precision. Biological samples were then diluted in PBS to reach the optimal particle count per frame in scatter mode (50–200 particles) and analyzed throughout 11 positions of the cell, with camera sensitivity 85, shutter speed 100, high video quality (capturing 60 frames) at 30 frames/s (1 cycle), minimal area 10, maximal area 1000 and brightness 25. For fluorescence measurements, a 500 nm cut-on long-pass filter was used with camera sensitivity adjusted to 95 and video quality to low (capturing 15 frames). “Low Bleach” technology was enabled during acquisition in order to reduce the laser exposure time for the fluorophore and capture fluorescence signal at its maximum intensity. 11-position tables and histograms were used in the data analysis.

### 4.5. Bulk Fluorescence Measurements

After labeling, 200 µL of fluorescent sEV samples were loaded in black 96-well plates (PerkinElmer, Waltham, MA, USA). Plates were inserted in a fluorometer plate reader (CLARIOstar Plus; BMG Labtech, Ortenberg, Germany) and for each fluorescence measurement, optimal gain and focal height were adjusted to the brightest well. Raw fluorescent signals of samples were normalized to a blank (PBS) and data was presented as signal-to-noise.

### 4.6. Statistical Analysis

Experiments were performed in triplicate unless stated otherwise. All results are presented as average with SEM. GraphPad Prism 9 (San Diego, CA, USA) and Two-way ANOVA with Tukey test for multiple comparisons were used to examine inter-batch (batch #A and batch #B) and cross-platform (nFCM and F-NTA) variability in tetraspanin expression, as well as the correlation between purified and non-purified samples.

## 5. Conclusions

This work addresses some of the main benefits and limitations of working with two different single-nanoparticle profiling platforms. We propose protocols for optimal sEV characterization on both instruments, but also raise concerns regarding the value of certain dyes, affinity reagents, or methodologies commonly employed. Additionally, composite sEVs characterization addressed in our study represents an important information feed for downstream applications, such as uptake studies, in which the importance of parameters such as size, aggregation status, maintained integrity of sEVs, as well as surface display of molecules that actively mediate the cell uptake (e.g., tetraspanins), has been proven fundamental for correct experimental design (i.e., EV dosage) and results elaboration. Hereby used analytical protocols consume a very small sample fraction, thus leaving a majority of well characterized sEV isolates intact for further testing and use. Throughout this report, we aimed to highlight the aspects and considerations that are often understated but highly important, where a balance between obtaining trustworthy data and pushing instruments to operate at the edges of current technological limits must exist. It is fundamental to underline that a critical attitude must drive experimental works and reports aimed at accurately dissecting the EV field, where irrefutable evidence over major topics is still lacking.

## Supplementary Materials

The following are available online at https://www.mdpi.com/article/10.3390/ijms221910510/s1.
