# Correction: Fortunato et al. Opportunities and Pitfalls of Fluorescent Labeling Methodologies for Extracellular Vesicle Profiling on High-Resolution Single-Particle Platforms. *Int. J. Mol. Sci.* 2021, *22*, 10510

**DOI:** 10.3390/ijms27073199

**Published:** 2026-04-01

**Authors:** Diogo Fortunato, Danilo Mladenović, Mattia Criscuoli, Francesca Loria, Kadi-Liis Veiman, Davide Zocco, Kairi Koort, Natasa Zarovni

**Affiliations:** 1 Exosomics SpA, 53100 Siena, Italy; dfortunato@exosomics.eu (D.F.); mcriscuoli@exosomics.eu (M.C.); dzocco@exosomics.eu (D.Z.); 2 HansaBioMed Life Sciences Ltd., 12618 Tallinn, Estonia; danilo@hansabiomed.eu (D.M.); francesca@hansabiomed.eu (F.L.); kadi-liis@hansabiomed.eu (K.-L.V.); 3 School of Natural Sciences and Health, Tallinn University, 10120 Tallinn, Estonia; kairi.koort@tlu.ee; 4 Department of Chemistry and Biotechnology, Tallinn University of Technology, 12618 Tallinn, Estonia; 5 Cell and Gene Therapy Research and Development, Lonza Inc., Rockville, MD 20850, USA

**Additional Affiliation(s)**

In the original publication [1], there was an error regarding the affiliation for Francesca Loria. In addition to Affiliation 2, the updated affiliation should include the following: Department of Chemistry and Biotechnology, Tallinn University of Technology, 12618 Tallinn, Estonia. The authors state that the scientific conclusions are unaffected. This correction was approved by the Academic Editor. The original publication has also been updated.
