# Supplementary material for: Opportunities and Pitfalls of Fluorescent Labeling Methodologies for Extracellular Vesicle Profiling on High-Resolution Single-Particle Platforms
Source: Int J Mol Sci. 2021 Sep 29;22(19):10510. doi: 10.3390/ijms221910510 (PMC8508895; doi:10.3390/ijms221910510)
Supplement: Supplementary file 1 [file ijms-22-10510-s001.zip › Supplementary figures.pdf]

nFCM controls

Blank reaction	Average conc. Fluo (p/uL)	SEM
CFSE	3.69E+03	1.05E+03
CMG	4.39E+04	7.35E+03
CTR	3.69E+02	5.22E+02
CMR	3.69E+02	5.22E+02

Blank reaction	Average conc. Fluo (p/uL)	SEM
CD81-PE	4.03E+02	7.45E+01
CD9-PE	6.62E+02	1.39E+02
CD63-PE	7.43E+02	3.20E+02

Blank reaction	Average conc. Fluo (p/uL)	SEM
CD81-488	9.20E+02	1.02E+02
CD9-488	2.34E+03	5.33E+02
CD63-488	8.18E+03	3.86E+03

F-NTA controls

Blank reaction	Average number of fluorescent particles per position	SEM
CFSE	1.75	0.55
CMG	1.77	0.63
Unstained	2.58	0.57

Blank reaction	Average number of fluorescent particles per position	SEM
CD81-AF488	0.95	0.05
CD9-AF488	1.50	0.10
CD63-AF488	0.85	0.15
Unstained	0.60	0.20

Blank reaction	Average number of fluorescent particles per position	SEM
CD9/CD81	1.40	-
CD9/CD63	2.10	-
CD81/CD63	0.65	0.05
CD9/CD81/CD63	2.00	0.80

F-NTA: Antibody staining PSD

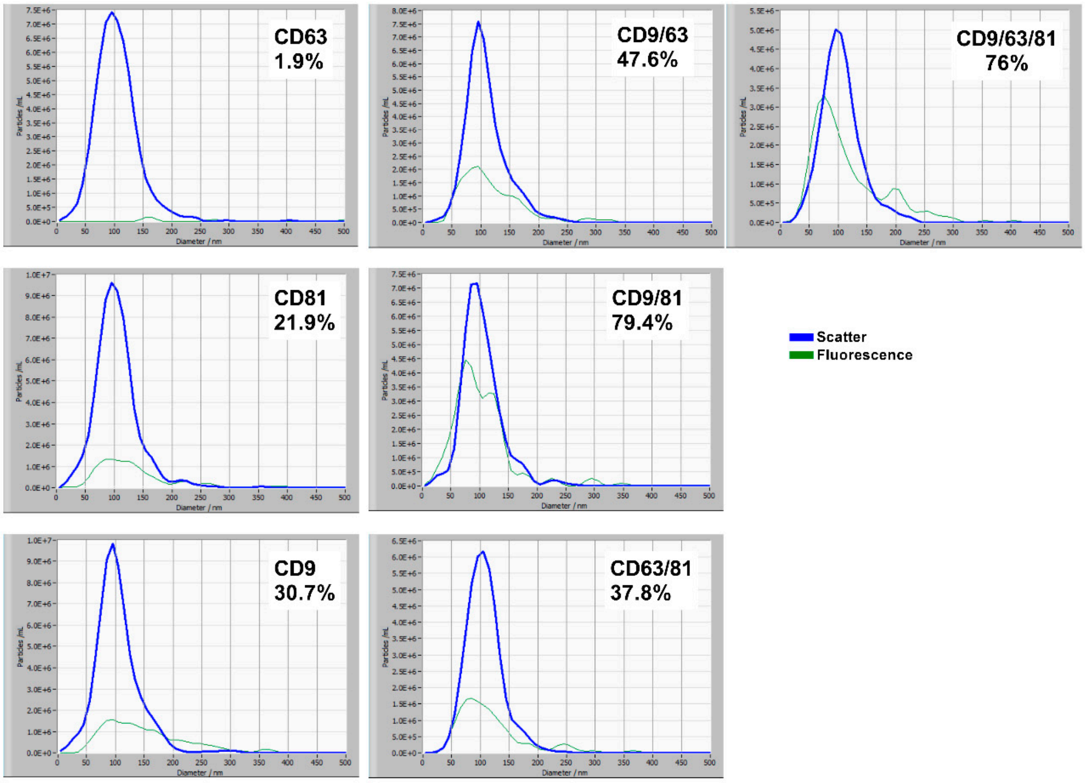

Supplementary Figure S1. Procedural controls and PSD histograms of antibody-stained sEVs. Blank (dye + buffer) and unstained reactions (sEVs + buffer) were used as controls in experiments. None of the membrane (CMG, CMR), cytoplasmic (CFSE, CTR) nor antibody labels (anti-CD9, anti-CD63 and anti-CD81; single, double and triple) showed fluorescent artefacts on nFCM and F-NTA, proving efficient dye removal prior to measurements. PSD histograms represent concentration and size of particles measured in scatter and fluorescence mode on F-NTA after single, double and triple staining with different anti-tetraspanin antibodies conjugated to AF488. Such results showed lower staining percentage with single antibodies, while PSD of fluorescent particles leaned more toward higher values. Double and triple staining reactions labelled more sEVs, with apparently smaller diameter particles being more visible in fluorescence mode.

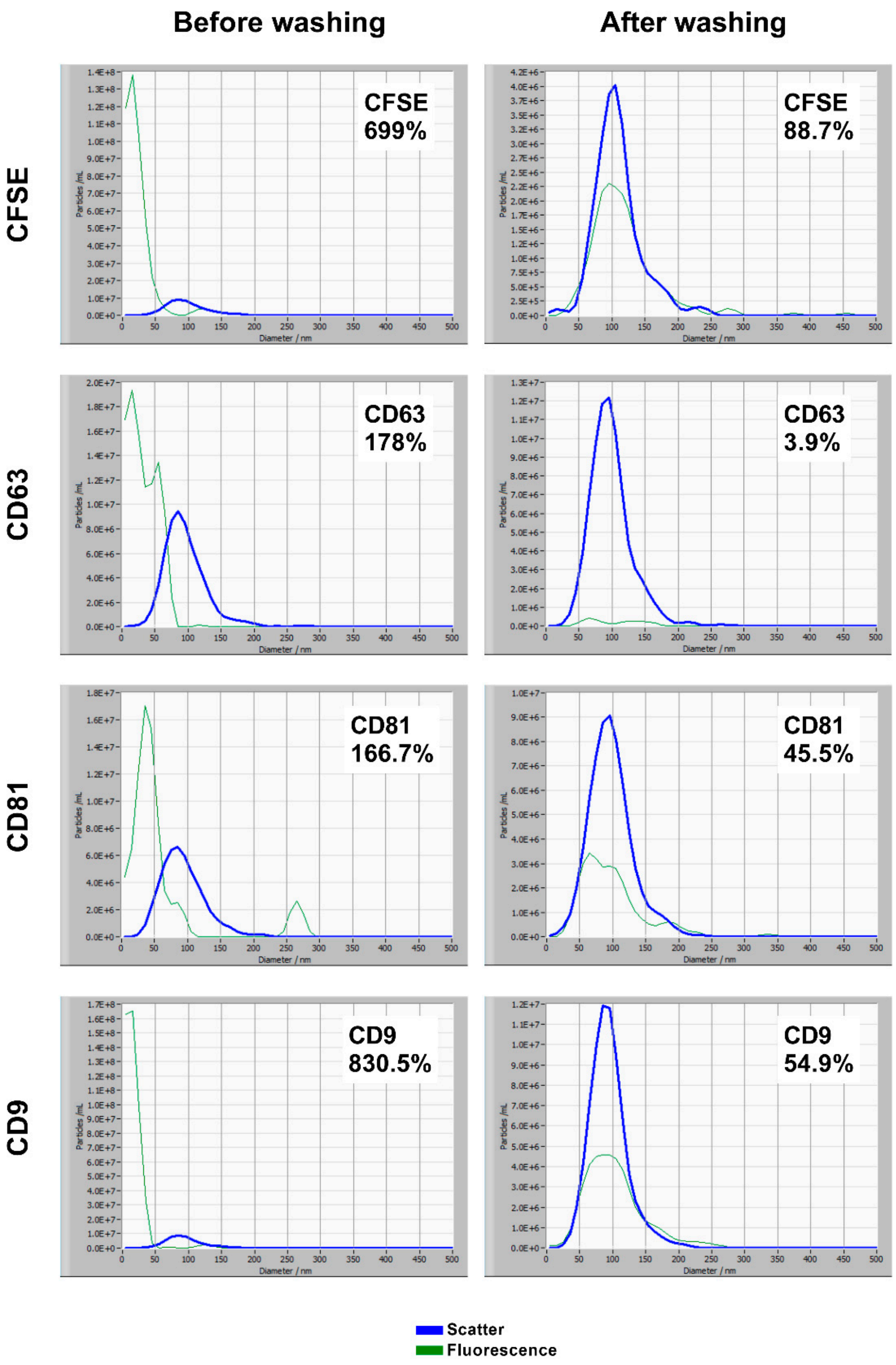

Supplementary Figure S2. Effect of excess dye on fluorescence measurements. Stained sEVs analyzed before UF washing showed very high fluorescent background, which interfered with accurate analysis of true positive events. UF efficiently removed all of the unbound dye, enabling proper fluorescence measurement, while maintaining total sEV subpopulation composition (scatter PSD before and after washing remains unchanged).

nFCM controls

Blank reaction	Average conc. Fluo (p/uL)	SEM
Ribogreen	1.87E+05	9.27E+04
Syto	4.71E+05	9.20E+04

F-NTA controls

Blank reaction	Average number of fluorescent particles per position	SEM
Ribogreen [1:2]	0.30	-
Syto [100µM]	0.90	-
Unstained	0.50	-

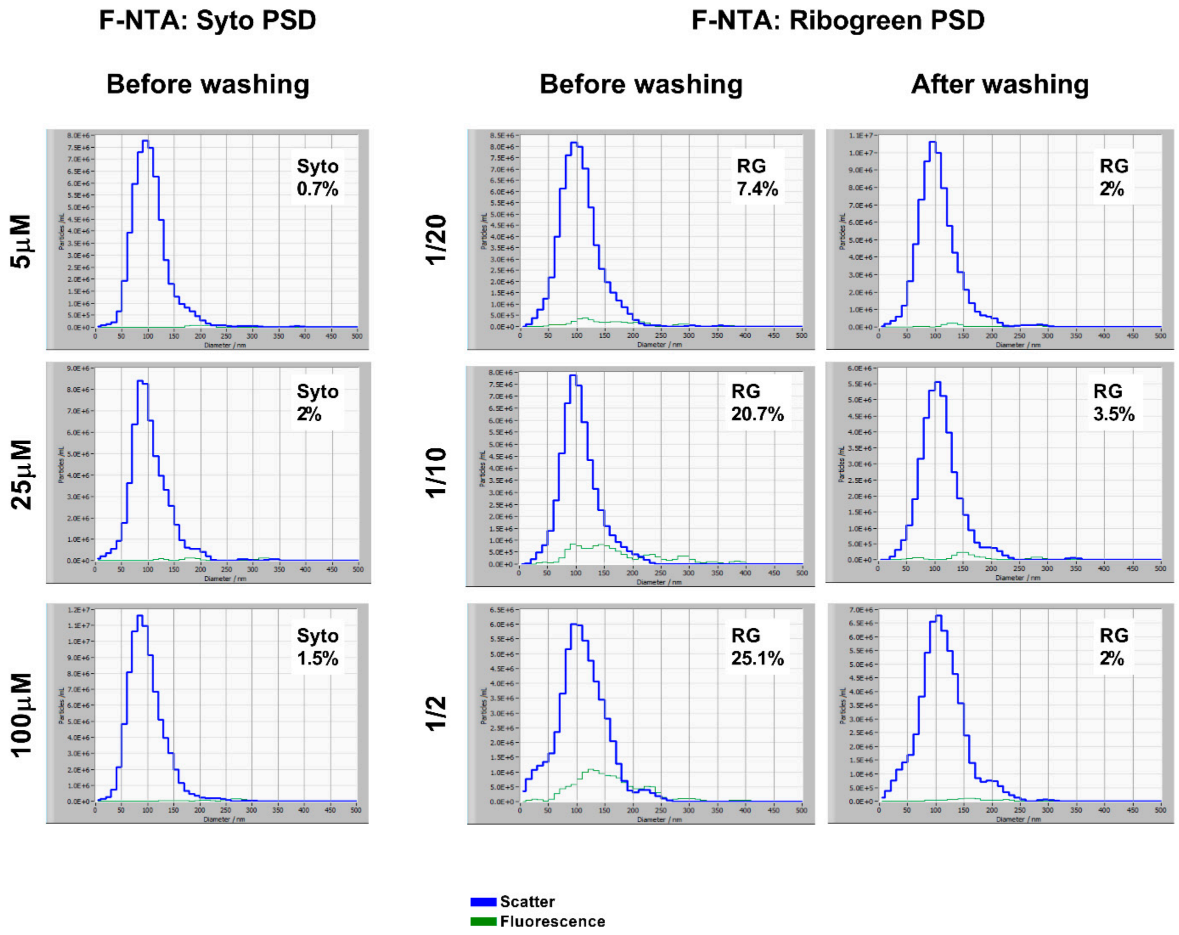

Supplementary Figure S3. Procedural controls and PSD histograms of sEVs stained with RNA dyes. Blank controls for both Syto and Ribogreen expressed negligible number of fluorescent events even without UF washing, showing that the dye on its own did not create artefacts. Reactions with Syto resulted in very low staining efficiency even with the highest concentration of 100µM. On the other hand, Ribogreen had a dose dependent increase in signal, with PSD showing preferential dye loading into larger particles. Nevertheless, signal was completely removed with UF washing.

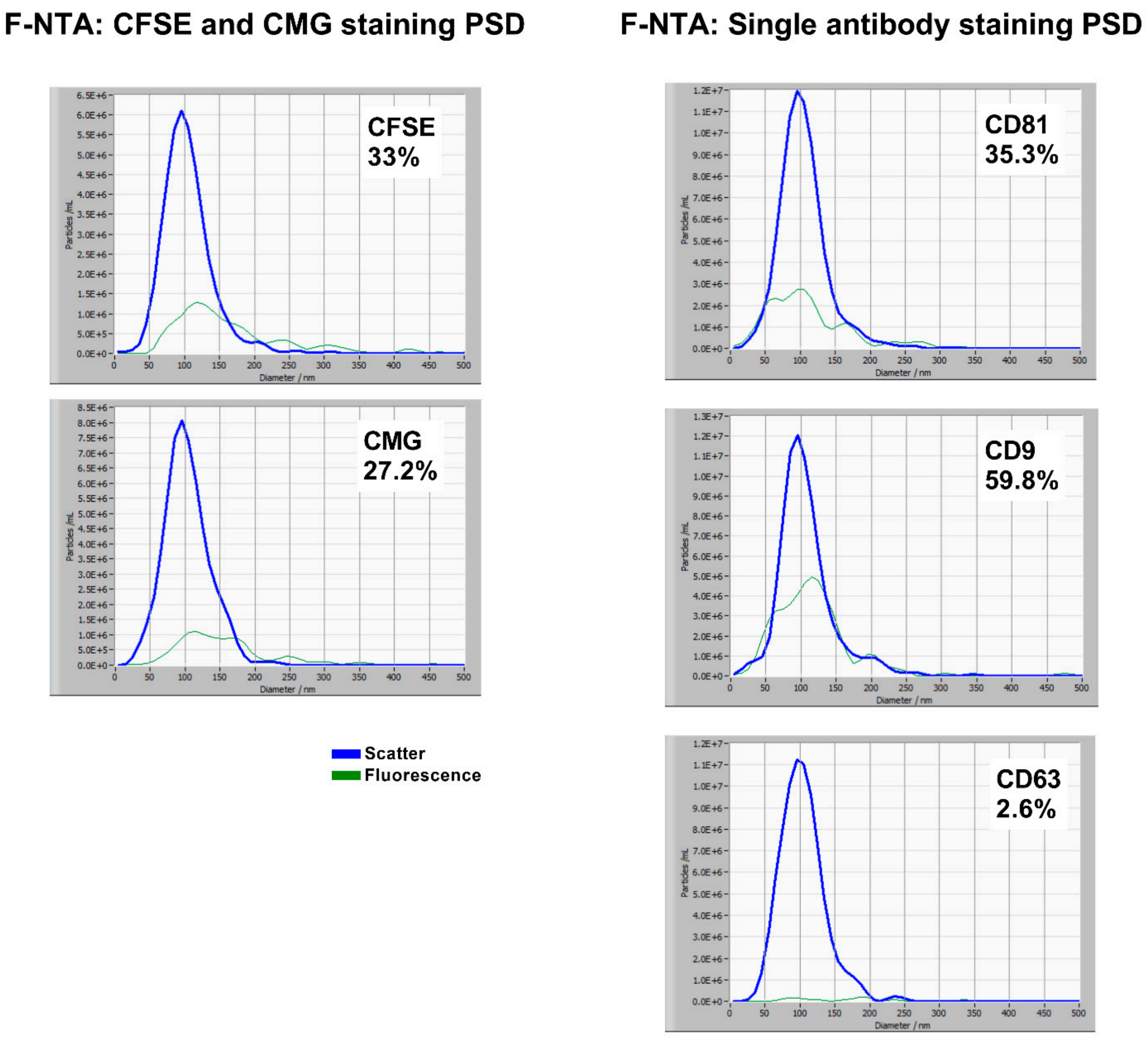

Supplementary Figure S4. F-NTA PSDs of sEVs stained directly in cell conditioned media. Staining of non-purified sEVs gave lower percentage of labelling for CFSE and CMG, with fluorescent PSD shifting to the right. However, single-antibody labelling had higher number of fluorescent events, compared to analogous reactions with purified sEVs.

nFCM: thyroglobulin control

	Thyroglobulin staining			
	Staining reaction	Average conc. Fluo (p/uL)	% staining	% SEM
PE	CD9	1.16E+03	0.55	0.05
	CD63	1.05E+03	0.35	0.05
	CD81	2.25E+03	0.65	0.15
AF488	CD9	4.21E+03	2.10	0.00
	CD63	4.58E+04	12.05	3.85
	CD81	2.44E+04	4.25	1.65

F-NTA: thyroglobulin control

Thyroglobulin staining			
Staining reaction	Average number of fluorescent particles per position	% staining	% SEM
CFSE	1449.40	1265.75	2.15
CMG	15.20	13.13	0.63

F-NTA: BSA control

BSA staining		
Staining reaction	Average number of fluorescent particles per position	SEM
Before washing	1404.7	155.0
After washing	1.1	0.2

Blank reaction	Average number of fluorescent particles per position	SEM
Before washing	0.6	0.1
After washing	0.4	0.0

Supplementary Figure S5. Procedural controls of dye specificity. Membrane and cytoplasmic dyes, as well as antibodies were tested for their specificity using large globular protein, thyroglobulin. While majority of antibodies (except for anti-CD63 conjugated to AF488) showed very high specificity, CFSE exerted strong non-specific activation in presence of other proteins. In case of thyroglobulin, activated CFSE was detectable even after UF washing, as the protein was large enough to remain in the UF retentate. This was not the case with BSA, where all of the activated CFSE was successfully washed out. Spontaneous activation of CFSE in buffer only (blank reactions) was not observed.

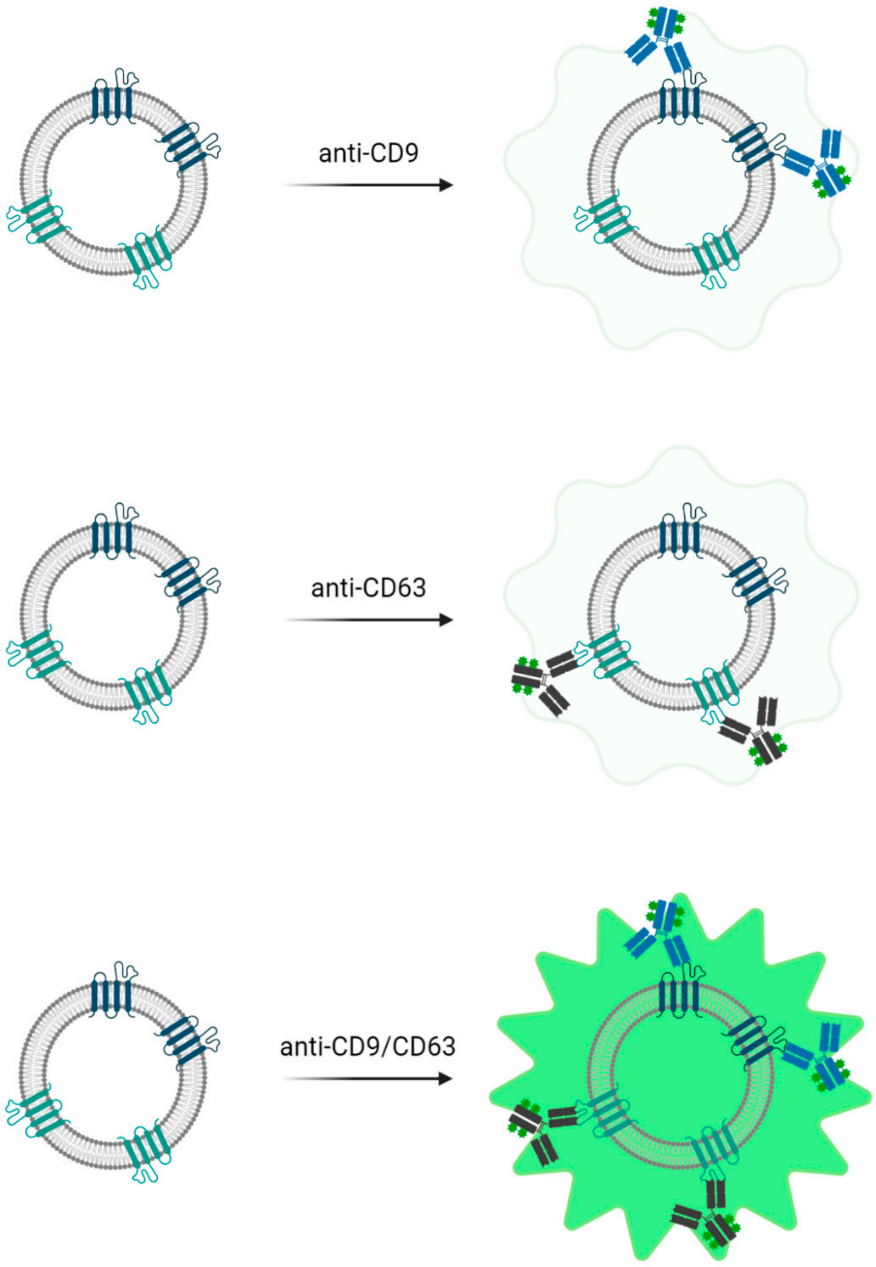

Supplementary Figure S6. Schematic representation (created on BioRender.com) of sEV staining by fluorescently labeled primary antibodies, proposing that epitope scarcity on the surface of sEVs may limit the complete detection of certain subpopulations upon single staining, due to a lack of sensitivity of the platform used. If the labeling of multiple tetraspanins with the same dye renders visible previously undetected events after single staining, a correct co-expression analysis is not possible due to an underestimation of single marker expression.
