# Supplementary material for: Opportunities and Pitfalls of Fluorescent Labeling Methodologies for Extracellular Vesicle Profiling on High-Resolution Single-Particle Platforms
Source: Int J Mol Sci. 2021 Sep 29;22(19):10510. doi: 10.3390/ijms221910510 (PMC8508895; doi:10.3390/ijms221910510)
Supplement: Supplementary file 1 [file ijms-22-10510-s001.zip › Supplementary table.pdf]

Table S1. Fluorescence properties of different fluorophores.

**Fluorophore**	**Extinction coefficient (ε) [$M^{-1}cm^{-1}$]**	**Quantum yield (Φ)**	**Brightness (ε × Φ)**
AF488	73,000	0.92	67,160
AF647	270,000	0.33	89,100
APC	700,000	0.68	476,000
PE	1,960,000	0.84	1,646,400
